# Dietary energy levels alter production performance, egg quality, and intestinal health of Wenshui green-shell layers in the peak laying period

**DOI:** 10.3389/fvets.2025.1735823

**Published:** 2025-12-15

**Authors:** Wenjing Liu, Yanjiao Di, Qikun Lu, Yu Jiang, Xuejun Yuan, Shuzhen Jiang, Weiren Yang, Ning Jiao

**Affiliations:** 1 Key Laboratory of Efficient Utilization of Non-Grain Feed Resources (Co-Construction by Ministry and Province), College of Animal Science and Technology, Ministry of Agriculture and Rural Affairs, Shandong Agricultural University, Tai’an, China; 2 College of Life Sciences, Shandong Agricultural University, Tai’an, China

**Keywords:** Wenshui green-shell layers, appropriate metabolizable energy, production performance, egg quality, intestinal microbiota

## Abstract

**Introduction:**

The Wenshui green-shell layers is a new layer breed, and the appropriate metabolizable energy (ME) have not been identified as the lack of breed-specific feeding standards. Therefore, this study aimed to investigate the effects of dietary energy levels on production performance, egg quality and intestinal health of Wenshui green-shell layers to identify appropriate ME in the peak laying period.

**Methods:**

A total of 600 33-week-old Wenshui green-shell layers were selected and randomly assigned to five treatment groups, with eight replicates of 15 laying hens each. The hens were fed with diets with ME level of 2,400 (ME2400), 2,500 (ME2500), 2,600 (ME2600), 2,700 (ME2700) and 2,800 (ME2800) kcal/kg for 42 days.

**Results and discussion:**

The results showed that the production performance of layers showed a quadratic curvilinear change with ME levels increase, and the ME2700 group obtained the optimal production performance and dry matter apparent availability. The serum albumin and triglyceride contents were linearly increased with the ME levels increase, while serum total antioxidant capacity of the ME2700 group was significantly higher than that in other groups. Additionally, egg quality was showed the quadratic curvilinear improvement, while the nutrient contents in eggs and superoxide dismutase activity in egg albumen were increased with increasing ME levels. Besides, the ME2700 group had a better morphology in oviduct and small intestine. Energy levels regulated the intestinal microbiota composition, notably ME2700 increased the relative abundance of *Rikenellaceae_RC9_gut_group*. On the other hand, the abundance of *Bacteroides* was negatively correlated with egg and albumen weight, while that of *Fusobacterium* was positively correlated with egg yolk ratio. In conclusion, the appropriate dietary ME level was 2,700 kcal/kg, which could improve production performance, egg quality and intestinal health in Wenshui green-shell layers during peak laying period.

## 1 Introduction

Dietary metabolizable energy (ME) serves as the most critical nutritional factor regulating production performance and affecting feed cost in poultry (1). Low energy levels impaired normal growth and production performance, whereas high energy level impaired normal intestinal development and production performance, and resulted in feed inefficiency resource inefficiency (2–5). Previous study showed that appropriate dietary ME level enhanced daily weight gain and laying rate while reduced feed conversion rate (6). Therefore, appropriate dietary energy intake could improve nutrient utilization, intestinal microorganism diversity, and intestinal health, thereby reducing production costs in the poultry industry (7). Therefore, it is necessary to precisely regulate energy supply to optimize poultry production performance.

Numerous studies have shown that dietary energy levels could affect the egg quality. It has been reported that yolk color (YC) increased, while the eggshell thickness decreased as dietary energy levels increased from 2,650 to 2,750 kcal/kg in Fengda-1 layers (8). Study showed that YC increased with ME from 2,400 to 2,550 kcal/kg, while further increased ME (2,700 to 2,850 kcal/kg) did not improve YC in Taihe Silky Fowl (9). Additionally, it has been reported egg weight increased as dietary energy level increasing from 2,700 to 2,800 kcal/kg of Hy-Line Brown laying hens in the middle stage of egg production (10). Thus, the effects of dietary energy levels on egg quality varied as the different breeds and laying stages of layer.

The ME requirement for laying hens is 2,900 kcal/kg at laying period (NRC, 1994). Whereas, the energy levels were recommended was 2,800 kcal/kg for Hyline layers and 2,700 kcal/kg for Lumane layers by their respective companies. However, Wenshui green-shell layers, a local specialty breed of chicken, is an improved breed crossed from the Wenshang reed-feather chicken and Xinyang green-shell layers, whose major characteristics are reed feathers, green-shelled eggs, a high egg-laying number, and excellent egg quality (11). Whereas, the nutritional standards for Wenshui green-shell layers are currently limited, which is also lack of ME requirement.

Currently, research on the appropriate dietary ME level and effects of ME level on production performance and egg quality in Wenshui green-shell layers is limited, hindering their productivity and economic potential. Therefore, this study aims to investigate the effects of dietary ME level on production performance, egg quality and intestinal microbiota of Wenshui green-shell layers to identify the appropriate dietary ME level in the peak laying period.

## 2 Materials and methods

### 2.1 Hens and dietary treatment

The Animal Care and Use Committee of Shandong Agricultural University (Shandong, China) approved the animal experiment (SDAUA-2021-081). The dietary energy gradients and CP levels were determined according to the Chinese Agricultural Standard (NY/T 33-2004). A total of 600 laying hens (aged 33 weeks) of Wenshui Green-Shell (Jinqiu Agricultural and Animal Husbandry Science and Technology Co., Ltd., Shandong, China) with similar body weight were randomly divided into five groups, each group with 8 replicates of 15 hens per replicate. The groups were designed as ME2400, ME2500, ME2600, ME2700 and ME2800 with ME levels of 2,400, 2,500, 2,600, 2,700 and 2,800 kcal/kg, respectively. The experimental design consisted of 7 days pre-feeding phase followed by 42 days formal trial period. The detailed formulation and nutritional composition of the diets were shown in Table 1.

**Table 1:** Ingredients and nutrient levels of the basal diet (air-dry basis, %).

Items	ME2400	ME2500	ME2600	ME2700	ME2800
Corn	50.66	53.34	57.94	60.17	57.64
Soybean meal	21.45	22.35	23.56	24.45	24.80
Wheat bran	15.80	11.50	5.40	1.46	1.80
Soybean oil	0.10	0.80	1.00	1.80	3.68
Limestone	8.78	8.64	8.54	8.55	8.52
CaHPO_3_	1.10	1.30	1.50	1.50	1.50
NaCl	0.10	0.06	0.06	0.06	0.05
Met	0.01	0.01		0.01	0.01
Premix^a^	2.00	2.00	2.00	2.00	2.00
Total	100	100	100	100	100
Nutrient levels					
Metabolic energy, kcal/kg	2,400	2,500	2,600	2,700	2,800
Crude protein	15.51	15.49	15.49	15.49	15.49
Methionine	0.35	0.35	0.35	0.36	0.36
Lysine	0.77	0.78	0.79	0.80	0.80
Tryptophan	0.20	0.20	0.20	0.20	0.20
Threonine	0.62	0.63	0.64	0.65	0.65
Argine	1.01	1.01	1.01	1.02	1.02
Ca	3.50	3.50	3.50	3.51	3.50
P	0.62	0.62	0.62	0.60	0.60

a The nutrient level is the calculated value, and the premix is provided for each kilogram of diet: VA, 11400 IU; VD_3_, 2,200 IU; VE, 75 mg; VK_3_, 6 mg; VB_1_, 3 mg; VB_2_, 16.5 mg; VB_6_, 6 mg; VB_12_, 0.03 mg; Fe, 80 mg; Cu, 20 mg; Mn, 120 mg; Zn, 110 mg.

Prior to the trial, the hen houses were thoroughly disinfected, followed by the maintenance of ambient temperature at 18–30°C and a daily light exposure of 16 h at 51 lux. Hens received ad libitum feed and water, and the weekly feed intake and residuals were accurately recorded. Eggs were collected, weighed, and counted daily at a fixed time. Flock health was monitored continuously, sick/dead chickens were removed in a timely manner, and flock information was updated in real time. Routine immunization procedures were followed during the trial.

### 2.2 Slaughter and sample collection

At the end of the experiment, eight eggs from each group were obtained to measure egg nutrients, another eight eggs from each group were obtained to measure egg antioxidant capacity, and then, three eggs from each replicate were obtained to measure egg quality. Six chickens from each group were randomly selected and euthanized by cervical dislocation at the end of the experimental trail. The cecal digesta were harvested and stored at −80°C for further analysis. In addition, the portions of the small intestine (duodenum, jejunum, ileum) and oviduct (magnum, uterine) were fixed in 4% paraformaldehyde solution for morphological analysis.

### 2.3 Measurement of production performance

The feed intake was recorded weekly for each replicate to calculate the average daily feed intake (ADFI). Additionally, egg number and weight were recorded daily for each replicate to calculate the average egg weight (AEW), average egg production rate (AEPR), average daily egg production (ADEP) and feed efficiency.


$$\begin{array}{l} \text{Average}\;\mathrm{egg}\;\text{weight}\;(g)=\text{Total}\;\mathrm{egg}\;\text{weight during the}\;\\ \text{statistical period}\;(g)/\text{Total number of eggs laid} \end{array}$$

$$\begin{array}{l} \text{Average}\;\mathrm{egg}\;\text{production rate}\;(\%)\\ =(\text{Total number of eggs laid during the statistical period})\\ /(\text{Number of laying hens}\times \text{Number of days})\times 100\% \end{array}$$

$$\begin{array}{l} \text{Average daily}\;\mathrm{egg}\;\text{production}\;(g)\\ =\text{Average}\;\mathrm{egg}\;\text{production rate}\\ \times \text{Average}\;\mathrm{egg}\;\text{weight} \end{array}$$

### 2.4 Determination of apparent metabolic rate of nutrients

At the 6th week of the experiment, digestive metabolism tests were conducted using metabolic cages for 4 days of pre-testing and 3 days of formal testing. The endogenous indicator method was used for the metabolic test. The daily feed intake of each chicken was measured and recorded precisely. Fecal samples were collected and weighed at regular intervals for three consecutive days, followed by nitrogen fixation using 10 mL of 10% sulfuric acid per 100 g of fresh feces. Subsequently, the fecal samples collected from each group were mixed, and the dry matter (DM), crude protein (CP), ether extract (EE), crude ash (Ash) and acid insoluble ash (AIA) in the manure samples and feeds were determined according to the Association of Official Agricultural Chemists (AOAC) (2012). The apparent metabolic rates of nutrients were calculated as followed:


$$\begin{array}{l} \text{Apparent metabolic rate of nutrient}\;(\%)\\ =[\begin{array}{l} 1-(\text{Feed}\;\mathrm{AIA}\;\text{content}/\text{Fecal}\;\mathrm{AIA}\;\text{content})\\ \times (\text{Feed nutrient content}/\text{Fecal nutrient content}) \end{array}]\times 100\% \end{array}$$

### 2.5 Assay of serum biochemistry and antioxidants

The concentrations of serum metabolites, including total protein (TP), albumin (ALB), urea nitrogen (UREA), glucose (GLU), triglyceride (TG), total cholesterol (T-CHO), high-density lipoprotein (HDL) and low-density lipoprotein (LDL) were measured using a COBUS MIRA Plus automatic biochemical analyzer (Roche Diagnostic System Inc., United States), following standard operating procedures. The antioxidant capacity of serum such as catalase (CAT), malondialdehyde (MDA), superoxide dismutase (SOD), total antioxidant capacity (T-AOC) and total glutathione (T-GSH) were measured using the microplate method in strict accordance with the instructions of the commercial kits (Nanjing Jiancheng Bio-Engineering Institute, China).

### 2.6 Measurement of egg quality, nutrients and antioxidant

At the end of the experiment, six eggs of each replicate were randomly selected to measure egg quality. Eggshell thickness (EST) was determined by eggshell thickness tester (ETG-5200, Robotmation, Japan). Egg length and egg width were measured using a vernier caliper and the egg shape index (ESI) was calculated by dividing the egg width by the egg length. Eggshell strength (ES) was measured using an eggshell strength tester (EFG-0503, Robotmation, Japan), with the egg positioned tip-down and blunt end-up. The height of albumen (AH), Haugh unit (HU) and yolk color (YC) were measured using a multi-functional egg tester (EMT-5200, Robotmation, Japan). The rate of yolk (YR) was determined as the yolk weight (YW) divided by the total egg weight (EW).

In addition, eggs were homogenized in separate petri dishes, weighed and labeled, followed by freeze-drying for 48 h and rehydration for 24 h to determine the DM content. The contents of CP, EE and Ash in eggs were then analyzed according to the method of AOAC (2012).

The antioxidant capacity of egg yolk and egg white, including CAT, MDA, SOD and T-GSH, were measured using the microplate method accordance with the instructions of the commercial kits (Nanjing Jiancheng Bio-Engineering Institute, China).

### 2.7 Morphological observation

At the end of the experimental period, the samples of small intestine (duodenum, jejunum, ileum) and oviduct (magnum, uterine) were collected for determination of morphology. Briefly, the fixed tissues were dehydrated in ethanol and xylene by gradient dehydration, embedded in paraffin and sliced into 5 μm thickness slices. The slices were stained with hematoxylin-eosin (HE) and sealed with neutral resin. The morphology was observed using a Nikon Elipse 80i microscope (Nikon, Japan) and the pictures were taken using a DP25 digital camera. Furthermore, the height of villi (VH) and the depth of crypts (CD) were measured from eight pictures per group and 40 crypts and villi per picture by an experimental researcher blinded to this experimental protocol. Then, the ratio of villi height to crypt depth (VH/CD) was calculated.

### 2.8 16S rRNA sequencing for cecal microbiota

Genomic DNA was extracted from cecal contents by CTAB method according to the manufacturer’s instructions, and the concentration and purity were checked by 1% agarose gel electrophoresis. The DNA samples were diluted to 1 ng/μL and the V3–V4 region of the bacterial 16S rRNA gene was amplified. The 16S rRNA gene was sequenced on the Illumina NovaSep platform according to the protocol by using the kits (Novogene. Co., Ltd., Beijing, China). The obtained sequences were rigorously filtered and screened, and the sequences were clustered into operational taxonomic units (OTUs) according to 97% concordance, and then, the valid data were subjected to OTUs clustering and species analysis. Alpha diversity analysis was performed using Quantitative Insight into Microbial Ecology to explore the diversity of cecal microflora. Beta diversity was assessed by calculating inter-sample distances using the Bray–Curtis index, which were then visualized via principal coordinate analysis (PCoA). The diversity, composition and variation of microbial communities were analyzed.

### 2.9 Statistical analysis

All data were analyzed using the general linear model (GLM) in SAS 9.4 statistical software (SAS Institute Inc., Cary, NC, United States), and differences among treatments were compared with Tukey’s multiple range tests. Spearman’s correlation analysis was used to evaluate the correlation between differential microbiota abundances and egg quality. Differences were considered significant at a *p*-value <0.05. Figures were drawn using GraphPad Prism 8.0 (La Jolla, CA, United States).

## 3 Results

### 3.1 Production performance

The effects of dietary energy levels on the production performance of Wenshui green-shell layers were shown in Table 2. The results showed that increasing dietary energy levels linearly reduced the ADFI in hens (*p* < 0.05). Both the AEPR and the ADEP exhibited a quadratic response, peaking in the ME2700 group, which showed the higher AEPR than the ME2400 and ME2800 groups, and the greater ADEP than the ME2400, ME2600 and ME2800 groups (*p* < 0.05). In contrast, there were no significant differences in the AEW and the feed efficiency among the groups (*p* > 0.05).

**Table 2:** Effects of dietary energy levels on production performance of Wenshui green-shell layers (*n* = 6).

Items	ME2400	ME2500	ME2600	ME2700	ME2800	SEM	*p*-value		
							Treatment	Linear	Quadratic
ADFI, g	91.12	91.69	90.92	89.97	88.53	0.627	0.113	0.013	0.024
AEPR, %	70.00^bc^	74.29^ab^	72.78^abc^	74.81^a^	68.54^c^	0.625	<0.001	0.597	0.001
AEW, g	47.87	48.11	48.41	48.83	48.61	0.151	0.280	0.037	0.095
ADEP, g	33.97^c^	35.81^ab^	34.40^bc^	36.62^a^	33.53^c^	0.261	<0.001	0.963	0.012
Feed efficiency	2.63	2.55	2.62	2.56	2.72	0.024	0.159	0.259	0.112

ADFI, average daily feed intake; AEPR, average egg production rate; AEW, average egg weight; ADEP, average daily egg production. ME2400, ME2500, ME2600, ME2700 and ME2800 indicated that the metabolizable energy level of the dietary was 2,400, 2,500, 2,600, 2,700 and 2,800 kcal/kg, respectively. Different shoulder mark letters of the same line are significantly different (*p* < 0.05). The tables below are the same.

### 3.2 Apparent metabolic rate of nutrients

The effects of dietary energy levels on the nutrient apparent metabolic rates of Wenshui green-shell layers were shown in Table 3. The DM metabolic rate in the ME2700 group was significantly higher than that in the ME2800 group (*p* < 0.05), whereas there were no significant differences on the metabolic rate of the CP, EE and Ash among the groups (*p* > 0.05).

**Table 3:** Effects of dietary energy levels on nutrient apparent metabolic rate of Wenshui green-shell layers (%, *n* = 6).

Items	ME2400	ME2500	ME2600	ME2700	ME2800	SEM	*p*-value		
							Treatment	Linear	Quadratic
DM	63.83^ab^	63.70^ab^	67.93^ab^	70.51^a^	60.38^b^	1.086	0.015	0.992	0.051
CP	42.67	42.40	44.48	47.92	39.51	1.684	0.645	0.948	0.590
EE	72.17	75.58	77.08	77.90	71.65	1.485	0.605	0.906	0.282
Ash	32.41	37.73	37.08	33.88	29.44	1.189	0.150	0.253	0.036

DM, dry matter; CP, crude protein; EE, ether extract; Ash, crude ash.

### 3.3 Blood indicators

#### 3.3.1 Serum biochemistry

The effects of dietary energy levels on the serum biochemistry indexes of Wenshui green-shell layers were shown in Table 4. Serum content of TP and ALB increased linearly with the increase of dietary energy levels, while the content of T-CHO in the ME2700 group was significantly higher than that in the ME2500 group (*p* < 0.05). However, dietary energy levels had no significant effects on the serum levels of TP, UREA, GLU, TG, HDL and LDL (*p* > 0.05).

**Table 4:** Effects of dietary energy levels on serum biochemical indexes of Wenshui green-shell layers (*n* = 6).

Items	ME2400	ME2500	ME2600	ME2700	ME2800	SEM	*p*-value		
							Treatment	Linear	Quadratic
TP, g/L	55.10	53.20	55.63	57.40	57.73	0.648	0.162	0.036	0.089
ALB, g/L	13.50^ab^	12.60^b^	12.55^b^	15.20^a^	15.33^a^	0.324	0.001	0.004	0.002
UREA, mmol/L	1.03	0.97	1.02	1.06	1.02	0.025	0.864	0.736	0.936
GLU, mmol/L	12.12	12.35	11.94	13.32	13.08	0.224	0.205	0.066	0.171
TG, mmol/L	6.73	6.59	6.72	6.42	6.35	0.144	0.903	0.368	0.656
T-CHO, mmol/L	2.34^ab^	2.25^b^	2.71^ab^	3.15^a^	2.66^ab^	0.105	0.039	0.035	0.069
HDL, mmol/L	0.77	0.72	0.83	0.86	0.76	0.026	0.465	0.460	0.577
LDL, mmol/L	0.70	0.63	0.71	0.87	0.75	0.035	0.277	0.172	0.401

TP, total protein; ALB, albumin; UREA, urea nitrogen; GLU, glucose; TG, triglycerides; T-CHO, total cholesterol; HDL, high-density lipoprotein; LDL, low-density lipoprotein.

#### 3.3.2 Serum antioxidant

The effects of dietary energy levels on the serum antioxidant indexes of Wenshui green-shell layers were shown in Table 5. The T-AOC level in the ME2700 group was significantly higher than that in the ME2400, ME2600 and ME2800 groups (*p* < 0.05). Whereas, dietary energy levels had no significant differences on the serum levels of CAT, MDA, SOD and T-GSH (*p* > 0.05).

**Table 5:** Effects of dietary energy levels on serum antioxidant indexes of Wenshui green-shell layers (*n* = 6).

Items	ME2400	ME2500	ME2600	ME2700	ME2800	SEM	*p*-value		
							Treatment	Linear	Quadratic
CAT, U/mL	13.44	13.93	17.64	17.05	15.76	1.148	0.741	0.260	0.439
MDA, nmol/mL	6.27	6.89	6.25	7.01	7.83	0.240	0.210	0.054	0.104
SOD, U/mL	103.54	108.45	110.08	116.54	112.83	2.731	0.684	0.174	0.351
T-AOC, mmol/L	0.77^b^	0.80^ab^	0.72^b^	0.99^a^	0.72^b^	0.028	0.003	0.720	0.589
T-GSH, μmol/L	2.92	2.73	2.31	2.34	2.27	0.133	0.452	0.069	0.164

CAT, catalase; MDA, malondialdehyde; SOD, superoxide dismutase, T-AOC, total antioxidant capacity; T-GSH, total glutathione.

### 3.4 Egg indicators

#### 3.4.1 Egg quality

The effects of dietary energy levels on the egg quality of Wenshui green-shell layers were shown in Table 6. The ES, AW and HU showed the quadratic curvilinear effects of increasing and then decreasing with the increase of dietary energy levels, and the ESI was linearly increased (*p* < 0.05). The ME2700 group showed the highest ESI and ES, whereas the ME2600 group had the greatest AW and HU (*p* < 0.05). In contrast, dietary energy levels had no significant effects on the EW, EST, YW, SW, AH, YC and YP (*p* > 0.05).

**Table 6:** Effects of dietary energy levels on egg quality of Wenshui green-shell layers (*n* = 6).

Items	ME2400	ME2500	ME2600	ME2700	ME2800	SEM	*p*-value		
							Treatment	Linear	Quadratic
EW/g	45.60	46.11	46.70	47.38	46.37	0.262	0.283	0.135	0.131
ESI	1.31^ab^	1.31^b^	1.31^b^	1.33^a^	1.32^ab^	0.003	0.011	0.033	0.068
EST/mm	0.33	0.33	0.33	0.34	0.33	0.003	0.840	0.571	0.794
ES/ (N/cm^2^)	38.04^b^	38.51^ab^	38.35^ab^	39.83^a^	39.22^ab^	0.209	0.037	0.011	0.039
YW/g	15.97	16.30	16.19	16.79	16.32	0.110	0.202	0.124	0.192
SW/g	6.00	6.23	6.13	6.16	6.06	0.046	0.594	0.875	0.411
AW/g	22.95^b^	22.67^b^	24.62^a^	23.34^ab^	22.71^b^	0.188	0.002	0.892	0.038
AH/mm	5.20	5.19	5.39	5.23	4.96	0.087	0.667	0.487	0.388
HU	75.91^ab^	76.48^ab^	77.48^a^	76.13^ab^	74.08^b^	0.384	0.070	0.139	0.016
YC	7.19	7.54	7.50	7.75	6.56	0.152	0.107	0.335	0.052
YR/%	35.01	35.35	34.69	35.55	35.27	0.287	0.908	0.733	0.938

EW, egg weight; ESI, egg shape index; EST, eggshell thickness; ES, eggshell strength; YW, yolk weight; SW, eggshell weight; AW, albumen weight; AH, albumen height; HU, Haugh unit; YC, yolk color; YP, percentage of yolk.

#### 3.4.2 Egg nutrient contents

The effects of dietary energy levels on the egg nutrient content of Wenshui green-shell layers were shown in Table 7. With the increase of dietary energy levels, the contents of DM, EE and Ash increased linearly in eggs (*p* < 0.05). The DM and the EE contents of the ME2800 group were significantly higher than those of the ME2600 and ME2500 groups, and the Ash content of the ME2800 group was significantly higher than those of the ME2400, ME2500 and ME2600 groups (*p* < 0.05). In contrast, dietary energy levels had no significant effect on the CP content in eggs (*p* > 0.05).

**Table 7:** Effects of dietary energy levels on egg nutrients of Wenshui green-shell layers (%, *n* = 6).

Items	ME2400	ME2500	ME2600	ME2700	ME2800	SEM	*p*-value		
							Treatment	Linear	Quadratic
DM	25.38^bc^	25.43^bc^	25.25^c^	26.23^ab^	26.80^a^	0.200	<0.001	<0.001	<0.001
CP	11.59	11.76	11.72	11.88	12.34	0.223	0.878	0.314	0.563
EE	9.27^ab^	9.26^ab^	8.84^b^	9.57^ab^	9.87^a^	0.109	0.023	0.046	0.014
Ash	0.92^b^	0.95^b^	0.93^b^	0.99^ab^	1.06^a^	0.013	<0.001	<0.001	<0.001

#### 3.4.3 Egg antioxidant

The effects of dietary energy levels on the antioxidant indexes of egg yolk and egg albumen in Wenshui green-shell layers were shown in Table 8, respectively. In terms of egg yolk antioxidant, dietary energy levels did not significantly affect the levels of CAT, MDA, SOD and T-GSH (*p* > 0.05). In terms of egg albumen antioxidant, SOD activity increased linearly with the increase of dietary energy levels (*p* < 0.05), whereas dietary energy levels had no significant effects on the levels of CAT, MDA and T-GSH in egg albumen (*p* > 0.05).

**Table 8:** Effects of dietary energy levels on egg antioxidation indexes in Wenshui green-shell layers (*n* = 6).

Items	ME2400	ME2500	ME2600	ME2700	ME2800	SEM	*p*-value		
							Treatment	Linear	Quadratic
Egg yolk									
CAT, U/mg prot	51.18	52.03	56.38	58.41	57.23	3.172	0.949	0.425	0.714
MDA, nmol/mL	184.60	180.56	205.30	199.42	211.44	6.617	0.565	0.124	0.317
SOD, U/mg prot	3.50	3.35	3.61	3.91	4.47	0.268	0.748	0.197	0.361
T-GSH, μmol/L	3.76	3.73	4.10	4.68	5.46	0.261	0.172	0.014	0.034
Egg albumen									
CAT, U/mg prot	36.98	37.80	32.21	36.80	32.61	2.230	0.913	0.551	0.842
MDA, nmol/mL	2.59	2.33	2.91	1.63	2.33	0.237	0.571	0.479	0.784
SOD, U/mg prot	1.19^b^	1.27^ab^	1.23^b^	1.28^ab^	1.37^a^	0.018	0.003	<0.001	0.003
T-GSH, μmol/L	0.97	0.90	0.69	0.93	1.13	0.049	0.065	0.324	0.026

### 3.5 Morphological observation

#### 3.5.1 Oviduct

**Figure 1:**
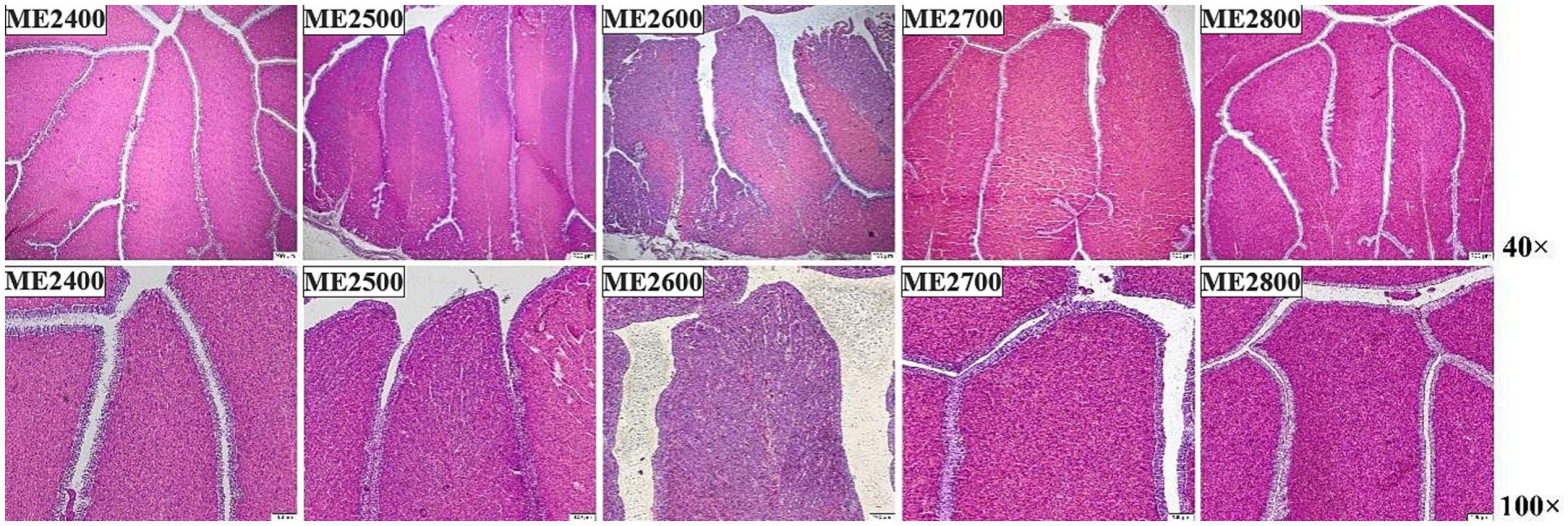
Effects of dietary energy levels on histological morphology of magnum of oviducts in Wenshui green-shell layers (*n* = 6). ME2400, ME2500, ME2600, ME2700 and ME2800 indicated that the metabolizable energy level of the dietary was 2,400, 2,500, 2,600, 2,700 and 2,800 kcal/kg, respectively. 40× means that the sectioned field of view was obtained under a 40 times optical microscope, 100× means that the sectioned field of view was obtained under a 100 times optical microscope. The picture below is the same.

**Figure 2:**
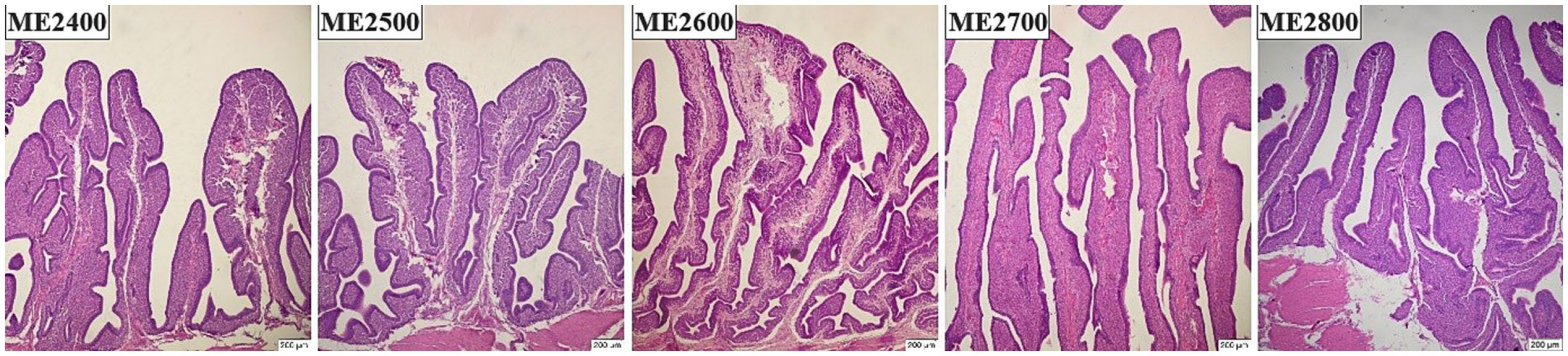
Effects of dietary energy levels on histological morphology of uterine of oviducts of Wenshui green-shell layers (*n* = 6). The section field was 40 times optical microscope.

The effects of dietary energy levels on the histological morphology of magnum and uterine of oviducts in Wenshui green-shell layers were shown in Figures 1, 2, respectively. The magnum of oviducts showed the well-defined mucosal epithelial lamina propria structure under low magnification, with vigorously secreting tubular glands and uniform mucus distribution in the interstitial tissue. The ME2700 group exhibited significantly thicker mucosal folds compared to other groups. High-magnification analysis further demonstrated higher glandular density in the lamina propria of the ME2700 group, whereas the ME2400 group showed reduced glandular density.

In addition, HE staining also showed good histological morphology of the uterine of oviducts with no obvious lesions as the dietary energy levels increased, and the height of the folds in the ME2700 group was significantly higher than in the other four groups, with more and tighter branching of the folds.

#### 3.5.2 Small intestine

**Figure 3:**
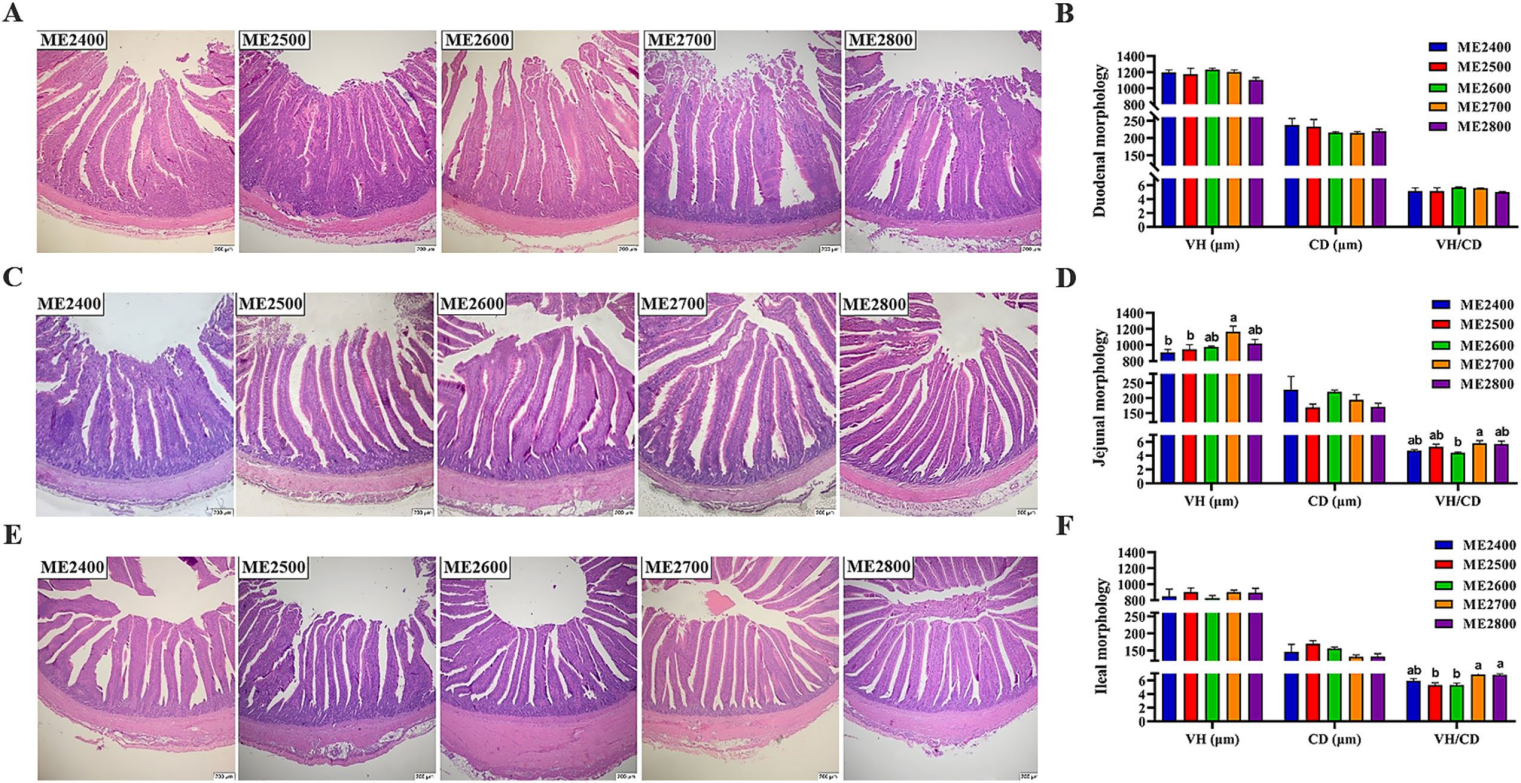
Effects of dietary energy levels on small intestinal histological morphology in Wenshui green-shell layers. **(A,C,E)** Representative hematoxylin and eosin staining images of duodenal, jejunal and ileal morphology, respectively. **(B,D,F)** Statistical analysis of **A,C,E**, respectively. The section field was 40 times optical microscope. *n* = 6. Results were presented as means ± SEMs.

The effects of dietary energy levels on the histological morphology of small intestine in Wenshui green-shell layers were shown in Figure 3. Dietary energy levels had no significant effects on the VH, CD and VH/CD of the duodenum (Figures 3A,B). However, the VH of the jejunum in the ME2700 group was significantly higher than that of the ME2400 and ME2500 groups, and its VH/CD was significantly higher than that of the ME2600 group (Figures 3C,D) (*p* < 0.05). The VH/CD of the ileum exhibited a linear increase in response to increased dietary energy levels, with the ME2500 and ME2600 groups demonstrating significantly lower values compared to the ME2700 and ME2800 groups (Figures 3E,F) (*p* < 0.05).

### 3.6 Cecal bacteria community

#### 3.6.1 Diversity

**Figure 4:**
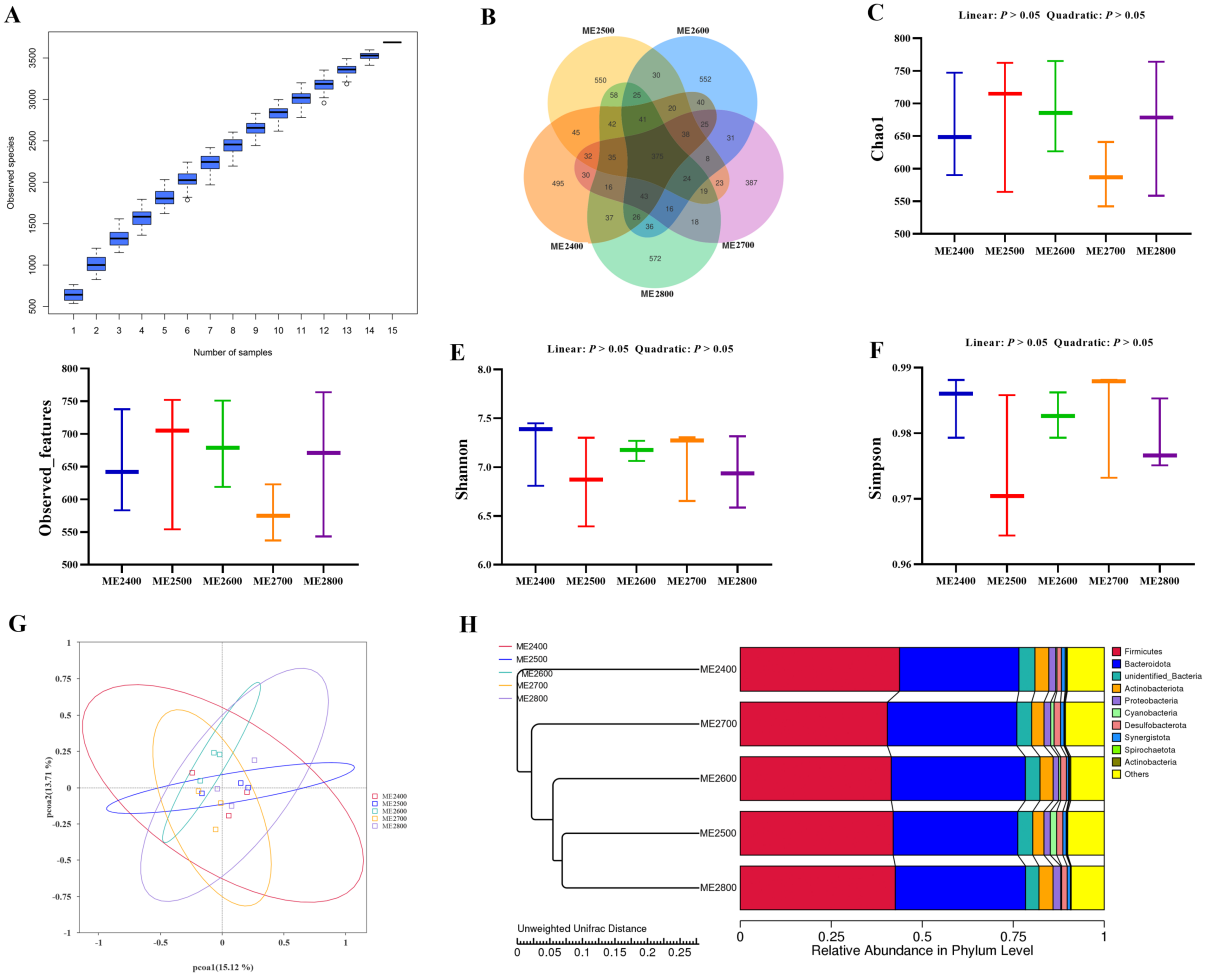
Effects of dietary energy levels on cecal microbial diversity and composition in Wenshui green-shell layers. **(A)** The species accumulation curve. **(B)** Venn diagram of number of operational taxonomic units (OUT). **(C)** Chao 1 index. **(D)** Observed_features. **(E)** Shannon index. **(F)** Simpson index. **(G)** Principal coordinate analysis (PCoA) based on Bray–Curtis distances. **(H)** Phylogenetic tree using bray_curtis algorithm. Results were presented as means ± SEMs. *n* = 3.

Effects of dietary energy levels on cecal microbial diversity and composition in Wenshui green-shell layers were shown in Figure 4. The species accumulation curve flattened out suggesting that the samples were sufficiently rich to predict sample species richness by OTU test (Figure 4A). A total of 1,340 OTUs were detected in the ME2400 group, 1,365 OTUs in the ME2500 group, 1,330 OTUs in the ME2600 group, 1,120 OTUs in the ME2700 group, and 1,383 OTUs in ME2800 group. The OTUs specific in ME2400, ME2500, ME2600, ME2700 and ME2800 groups were 495, 550, 552, 387 and 572, respectively. Moreover, all the groups shared 375 OTUs among their cecal microbiota (Figure 4B). However, dietary energy level did not affect cecal microbial diversity indicated by Chao 1 (Figure 4C), abserved_features (Figure 4D), Shannon (Figure 4E) and Simpson (Figure 4F) indexes. Additionally, the beta diversity presented by PCoA plot showed that the groups were clustered together (Figure 4G). The UPGMA phylogenetic tree showed that the ME2400 group was distributed in separate branches (Figure 4H).

#### 3.6.2 Bacterial abundance

**Figure 5:**
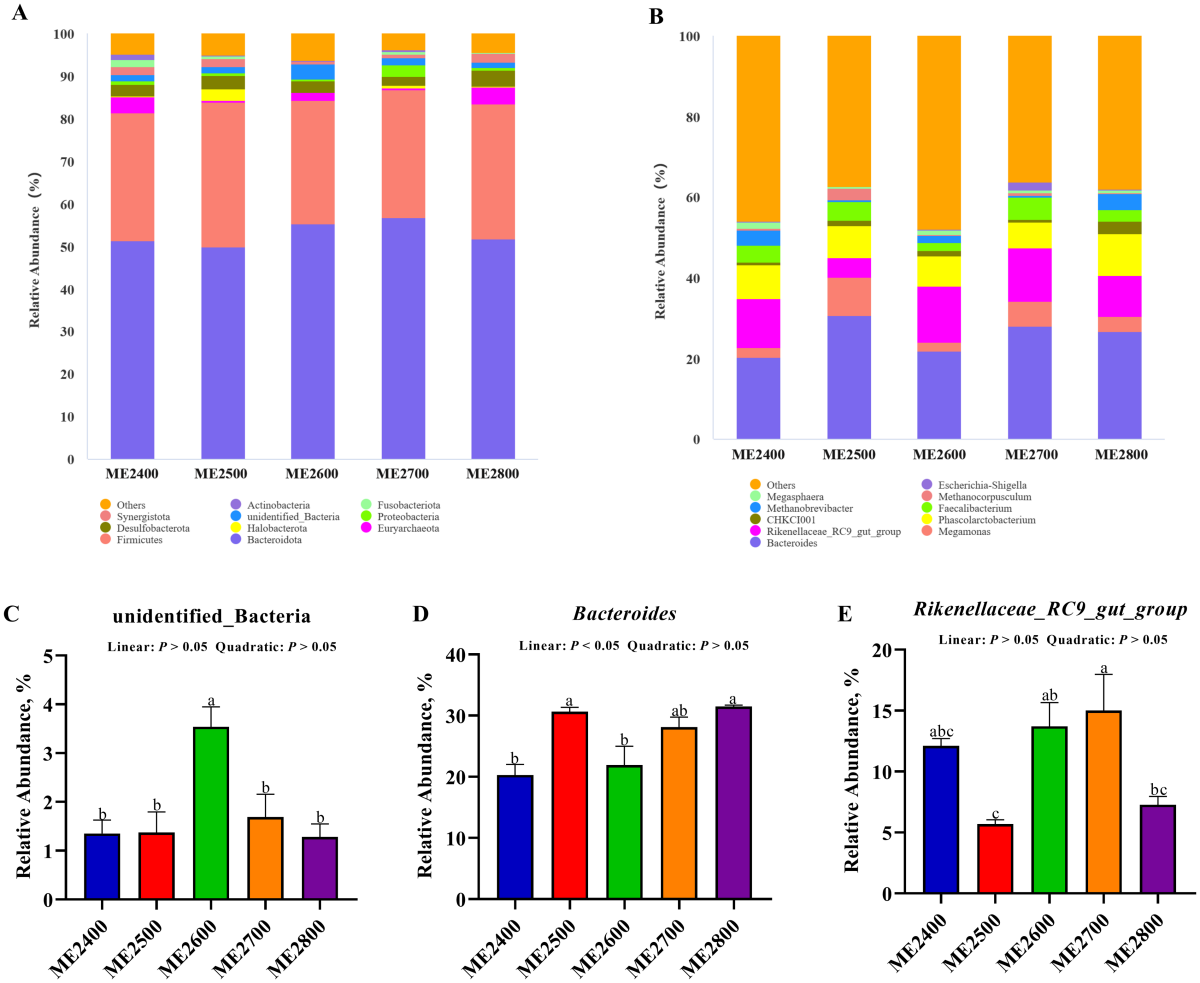
Effects of dietary energy levels on cecal microbial community at phylum and genus level in Wenshui green-shell layers. **(A)** Histogram of abundances of cecal microbiota at phylum level (top 10). **(C)** Abundance of unidentified_Bacteria. **(B)** Histogram of abundances of cecal microbiota at genus level (top 10). **(D)** Abundance of *Bacteroides*. **(E)** Abundance of *Rikenellaceae_RC9_gut_group*. Results were presented as means ± SEMs. *n* = 3. ^a–c^Represent different mark letters are significantly different (*p* < 0.05).

The abundances of top 10 microbial at phylum in cecal microbiota were shown in Figure 5A. The main dominant bacteria were Bacteroidota and Firmicutes. The abundances of Bacteroidota were 51.39, 49.82, 55.35, 56.76 and 51.86%, and those of Firmicutes were 29.99, 34.12, 28.79, 30.02, and 31.73% in the ME2400, ME2500, ME2600, ME2700 and ME2800 groups, respectively. As shown in Figure 5C, the relative abundance of unidentified_Bacteria in the ME2600 group was significantly higher than that in the other four groups (*p* < 0.05). The other microbial at the cecal microbial phylum was not different among the groups.

The abundances of top 10 bacterial at genus level in cecal microbiota were shown in Figure 5B. The main dominant bacteria were *Bacteroides* and *Rikenellaceae_RC9_gut_group*. The abundances of *Bacteroides* were 20.29, 30.65, 21.91, 28.11, and 31.47%, and those of *Rikenellaceae_RC9_gut_group* were 12.11, 5.69, 13.71, 15.00, and 7.27%in the ME2400, ME2500, ME2600, ME2700 and ME2800, respectively. As shown in Figure 5D, the abundance of *Bacteroides* in the ME2500 and ME2800 groups were significantly higher than that of the ME2400 and ME2600 groups (*p* < 0.05). Additionally, the abundance of *Rikenellaceae_RC9_gut_group* in ME2700 group was significantly higher than that of ME2500 and ME2800 groups (*p* < 0.05) (Figure 5E), while there was no significant difference in the remaining bacterial at genus level (*p* > 0.05).

#### 3.6.3 Relevance analysis

**Figure 6:**
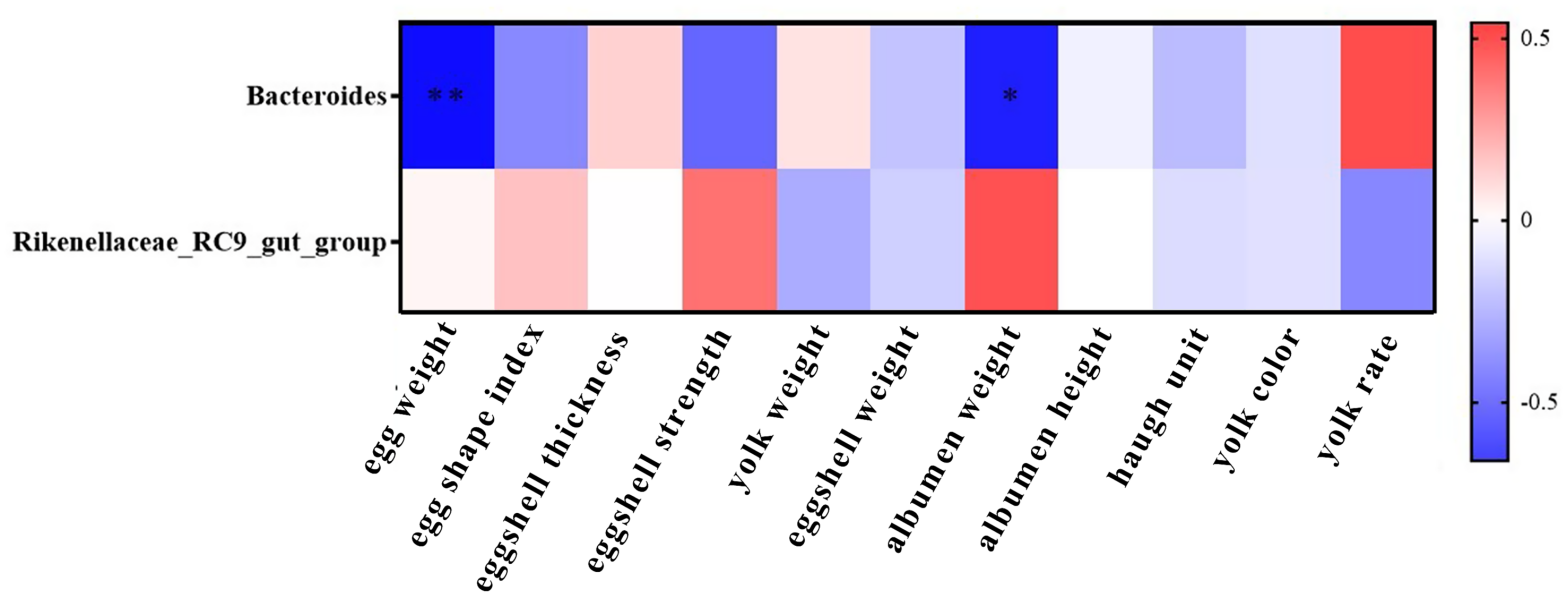
Heat map of correlation analysis between cecal microorganisms and egg quality indexes in ME groups of Wenshui green-shell layers. ^*^Represents *p* < 0.05 and ^**^represents *p* < 0.01.

The heat map of Spearman correlation analysis between cecal microorganisms and egg quality indexes in Wenshui green-shell layers were shown in Figure 6. In the experiment, we selected the top 10 genera with different relative abundance of cecal microbial community in ME groups for correlation analysis with the egg quality. The results showed the relative abundance of *Bacteroides* was negatively correlated with eggshell thickness (*p* < 0.05). In addition, the relative abundance of *Rikenellaceae_RC9_gut_group* was not collected with egg quality (*p* > 0.05).

## 4 Discussion

Energy is a key factor for poultry to maintain their survival and growth purposes. It has been reported high dietary energy level led to excessive body fat deposition, negatively affected production performance and health (12). Study has shown that feeding low-energy diets led to a decrease in egg quality, while feeding high-energy diets adversely affected the laying rate and reduced the production performance in layers (13). Consistently, this study found that with the increase of dietary energy levels, the AEPR and the ADEP of Wenshui green-shell layers showed a quadratic response. It indicated that dietary low energy level hindered the growth and development of laying hens, while dietary high energy level led to over fat and affected the laying performance. The study also found that ADFI of Wenshui green-shell layers decreased as the dietary energy levels increased. This aligned with previous study, which showed that at equal protein levels, the high-energy group had the lower ADFI than the low-energy group (10). The changes in production performance were associated with nutrient metabolism.

Previous study has reported that higher dietary energy increased the metabolic rate of EE, but had no significant effect on the metabolic rate of CP in poultry (14), whereas study found that increasing dietary metabolic energy from 2,630 to 2,940 kcal/kg enhanced the apparent metabolic rate of DM and EE in Taisheng pigeons (6). On the contrary, this study demonstrated that dietary energy levels significantly affected the DM metabolic rate of Wenshui green-shell layer. The ME2700 group had a significantly higher DM metabolic rate, while no significant differences were observed for CP, EE, or Ash metabolic rate. These variations might be due to differences in poultry breed, growth stage, or dietary energy levels.

The changes caused by dietary energy levels closely related the intestinal health. The VH, CD, and VH/CD ratio are important indicators for assessing absorption capacity and functional status of the intestine (15–17). This study found that as the dietary energy levels increased, the VH and VH/CD ratio of the jejunum in laying hens increased linearly, reaching a maximum at a metabolic energy level of 2,700 kcal/kg. And the VH/CD ratio of the ileum also showed a linear increasing trend with the increase of dietary energy levels. It was consistent with the present study, which found that decreasing dietary ME level significantly reduced the VH and VH/CD ratio in broiler jejunum (5). This indicated that dietary low energy prevented poultry from maintaining normal intestinal development, and then impaired production performance. Intestinal microbiota plays a crucial role in feed digestion and nutrient absorption, and supporting intestinal immune function by protecting against pathogenic infections, which abundance and species diversity is essential for understanding and promoting intestinal health (18). Research has found that Bacteroidetes and Firmicutes were the two most dominant phyla in the intestinal bacterial communities of poultry, playing a crucial role in maintaining intestinal microbial balance, and were classified as beneficial bacteria (19). This study revealed a consistent phylum-level advantage pattern, which showed Bacteroidetes and Firmicutes together constituted 80–87% of the cecal microbiota, consistent with previous report on the intestinal microbiota of poultry (20). Subsequently, we analyzed the composition of the microbiota at genus level and found that the dominant genera were *Bacteroides* and *Rikenellaceae_RC9_gut_group*, consistent with previous study (21). *Bacteroides* was a genus within the Bacteroidetes phylum that promoted the absorption of nutrients in the intestine and reduced the risk of obesity (22). The abundance of *Bacteroides* in the cecum was significantly higher in ME2500 and ME2800 groups compared to ME2400 and ME2600 groups. The abundance of *Bacteroides* did not show a clear trend with changes in dietary energy levels, which might be related to the substantial inter-sample variability. These findings suggested that moderately elevating dietary energy levels might increase the abundance of beneficial bacteria (23).

Blood serves as the carrier of metabolite in animals, and serum biochemical indexes can reflect the physiological health status and metabolic conditions of the organism (24). This study found that serum contents of ALB and T-CHO in Wenshui green-shell layers increased linearly with the increase of dietary energy levels. ALB not only has anti-inflammatory and antioxidant properties, but also can measure liver function (25). T-CHO refers to the sum of all cholesterol contained lipoproteins in blood, whose serum level can reflect lipid utilization (21). Previous study on quails showed that increasing dietary ME levels led to a rise in serum T-CHO content (26). And another study also showed that high dietary energy levels led to serum TP and T-CHO contents increasing in Taihe Silky Fowl during the peak laying period (21). The above results were consistent with our findings. We speculated that the elevated serum T-CHO content likely resulted from increased blood lipids, which were caused by high fat content in the high energy dietary.

Serum antioxidant serves as a helpful index for reflecting the oxidative stress status and health risks of the body. Livestock and poultry produce antioxidant enzymes like CAT and SOD, to decompose peroxides into less toxic or non-toxic substances through redox reactions, thereby protecting organism from oxidative damage (27, 28). T-AOC refers to the overall antioxidant level composed of various antioxidant substances and antioxidant enzymes. Study has shown that dietary supplementation with 2 and 4% palm oil significantly reduced MDA content in the serum of broilers while enhancing T-AOC level, compared to the 6% palm oil group (29). Similarly, we observed that the changes in dietary energy levels significantly affected the T-AOC level in the serum of laying hens, manifested as the T-AOC levels of the ME2700 group being greater, while having no significant effects on the levels of MDA, CAT and SOD. It is indicated that appropriately reducing the dietary energy levels could reduce the occurrence of lipid peroxidation and enhance the total antioxidant capacity in laying hens.

The thickness and toughness of the eggshell are key indicators for evaluating eggshell quality, both of which are closely related to egg breakage rates. A certain EST can significantly reduce egg breakage during transportation, while ES reflects the freshness and integrity of the egg (30). This study showed that dietary energy levels linearly enhanced ES, without affecting EST. Additionally, the egg geometry is also essential indicator of eggshell quality and hatchability, and is typically determined by ESI, as defined by the ratio of egg transverse diameter to longitudinal diameter (31). This study showed that ESI of laying hens increased linearly with increasing dietary energy levels. The quality of albumen is reflected in HU, which is expressed as a ratio of the thick albumen height and egg weight, whereas the higher HU scores indicate a better quality of albumen (32).

In this study, we observed that AW and HU increased first and then decreased with increasing dietary energy levels, reaching the highest levels at the ME2600 group, while AH was not affected by dietary energy levels. However, previous studies have shown that AH and HU were not affected by dietary energy levels (8, 33). However, study showed dietary ME levels had no effects on egg quality including AH, HU, YC, EST and ES of Taihe Silky Fowl during the peak laying period (21). The inconsistency might be attributed to the various growth stages and breeds of laying hens, leading to varying sensitivities to dietary energy levels. The specific mechanisms underlying required further investigation. In addition, we also found that the content of DM, EE, and Ash in eggs increased linearly with increasing dietary energy levels. Consistently, previous study showed that reduced dietary energy levels decreased the content of EE and CP in both egg yolk and albumen, with a significant reduction in YW (34). Since egg yolk was primarily composed of fat, we speculated that an increase in the proportion of yolk within the egg led to a rise in EE. These findings suggested that both excessively high and low dietary energy levels adversely affected egg quality. Under the experimental conditions of this study, the optimal egg quality for Wenshui green-shell layers was achieved with dietary energy level of 2,600 kcal/kg.

Eggs are a highly valued sourced of animal protein, providing a rich supply of essential amino acids and polyunsaturated fatty acids. However, these rich nutrients also make eggs highly susceptible to deterioration during storage, primarily manifested as protein denaturation and lipid peroxidation. Therefore, in is necessary to measure the antioxidant capacity of eggs (35). This study observed that changes in dietary energy levels had no significant effects on antioxidant indicators in egg yolks. Consistent with the previous study, dietary different energy levels had no significant effects on MDA content and free radical scavenging activity in egg yolks (36). This study also found that the level of SOD in albumen showed a linear increase with increasing dietary energy levels. Currently, there were no reports on the effects of dietary energy levels on albumen antioxidant indicators, and the mechanism by which dietary energy levels affecting egg antioxidant indicators also needed further investigation.

In sexually mature hens, the oviduct receives the ovum from the ovary and facilitates egg formation and potential fertilization (37). During egg formation, mature ovum passes successively through the infundibulum, magnum, isthmus, and uterine part of the oviduct to form substances such as albumen and eggshell, and then ultimately expelled through the cloaca. The magnum is where egg white forms, while the uterine serves as the position for eggshell formation and pigment deposition. The structural and morphological features of these regions directly determine the quality of both the albumen and eggshell (37, 38). Lu et al. (39) found that as dietary energy levels decreased, both the length and weight indices of the oviduct in 18–20 weeks old laying hens significantly decreased, indicating that overly low dietary energy levels were detrimental to the development of the oviduct in laying hens. Consistently, this study showed that as dietary energy levels increased, no pathological damage was observed in the magnum and uterine of the oviduct among all groups. Specifically, ME2600 and ME2700groups exhibited thicker folds and better morphology in the magnum than other groups, while ME2700 group had significantly higher fold height in the uterine compared to the other four groups, with ME2800 group having the lowest fold height in the uterine. This might be due to the high energy level in ME2800 group, which caused fat accumulation in laying hens and affected reproductive organ development, though specific mechanism required further study.

To further investigate potential functional implications, we conducted a Spearman correlation analysis between differentially abundant microbial genera in the cecum and egg quality parameters. Interestingly, the relative abundance of *Bacteroides* showed a negative correlation with EST. The underlying mechanism of this association remains unclear and requires further investigation.

## 5 Conclusion

This study showed that along with production performance, egg quality and intestinal health, the appropriate dietary energy level for Wenshui green-shell layers during the peak laying period was determined to be 2,700 kcal/kg.

## Data Availability

The datasets presented in this study can be found in online repositories. The names of the repository/repositories and accession number(s) can be found at: https://www.ncbi.nlm.nih.gov/, PRJNA1357030.

## Glossary

ADFI: Average daily feed intake
AEPR: Average egg production rate
AEW: Average egg weight
AH: Albumen height
ALB: Albumin
Ash: Crude ash
AW: Albumen weight
CAT: Catalase
CP: Crude protein
DM: Dry matter
EE: Ether extract
ES: Eggshell strength
ESI: Egg shape index
EST: Eggshell thickness
EW: Egg weight
GLU: Glucose
HDL: High density lipoprotein
HU: Haugh unit
LDL: Low density lipoprotein
MDA: Malondialdehyde
SOD: Superoxide dismutase
SW: Eggshell weight
T-AOC: Total antioxidant capacity
T-CHO: Total cholesterol
TG: Triglyceride
T-GSH: Total glutathione
TP: Total protein
UREA: Urea nitrogen
YC: Yolk color
YR: Yolk rate
YW: Yolk weight
